# Nano-encapsulated PCM via Pickering Emulsification

**DOI:** 10.1038/srep13357

**Published:** 2015-08-17

**Authors:** Xuezhen Wang, Lecheng Zhang, Yi-Hsien Yu, Lisi Jia, M. Sam Mannan, Ying Chen, Zhengdong Cheng

**Affiliations:** 1 Artie McFerrin Department of Chemical Engineering, Texas A&M University, College Station, TX, 7843-3122, USA; 2 Mary Kay O’Connor Process Safety Center, Artie McFerrin Department of Chemical Engineering, Texas A&M University, College Station, TX, 77843-3122, USA; 3 Department of Materials Science and Engineering, Texas A&M University, College Station, TX, 77843-3003, USA; 4 Soft Matter Center, Guangdong Province Key Laboratory on Functional Soft Condensed Matter, School of Materials and Energy, Guangdong University of Technology, Guangzhou, 510006, China; 5 Professional Program in Biotechnology, Texas A&M University, College Station, TX, 77843-3122, USA

## Abstract

We designed a two-step Pickering emulsification procedure to create nano-encapsulated phase changing materials (NEPCMs) using a method whose simplicity and low energy consumption suggest promise for scale-up and mass production. Surface-modified amphiphilic zirconium phosphate (ZrP) platelets were fabricated as the Pickering emulsifiers, nonadecane was chosen as the core phase change material (PCM), and polystyrene, the shell material. The resultant capsules were submicron in size with remarkable uniformity in size distribution, which has rarely been reported. Differential scanning calorimetry (DSC) characterization showed that the capsulation efficiency of NEPCMs, and they were found to be thermal stable, as characterized by the DSC data for the sample after 200 thermal cycles. NEPCMs exhibit superior mechanical stability and mobility when compared with the well-developed micro-encapsulated phase change materials (MEPCMs). NEPCMs find useful applications in thermal management, including micro-channel coolants; solar energy storage media; building temperature regulators; and thermal transfer fabrics.

Encapsulated phase change materials (PCMs) suspensions function as effective micro-channel coolants^1^, however, the encapsulated PCMs engineered to date, which measure several tens of microns, tend to fracture or clog the micro-channels due to their relatively large size^2^. Smaller encapsulated PCMs, such as NEPCMs, are rarely fabricated. Reducing the particle size of encapsulated PCMs enhances their mechanical stability and mobility. Moreover, NEPCMs present a larger total heat exchange area in comparison with conventional encapsulated PCMs, enhancing heat transfer rate between the coolant and micro devices, thus reducing mass flow rate and pump energy consumption. NEPCMs, therefore, are favorable as micro-channel coolants. NEPCMs also serve in other applications, including solar energy storage media^3^,^4^, building temperature regulators^5^, and thermal transfer fabrics^6^. To build up core-shell structures of NEPCMs, small droplets of the phase change materials core, such as paraffin, are dispersed in solvent. During emulsification, a stable nanoemulsion of paraffin is preferable.

In this work we are trying to optimize the emulsifying process by employing a Pickering emulsion procedure. Pickering emulsions are stabilized by colloidal particles^7^,^8^ where the apparent interfacial energy is reduced as solid particles are adsorbed onto the liquid-liquid interface, resulting in a stabilized emulsion. Previous studies proved that spherical, rod-like, and discotic particles can be used as Pickering stabilizers. Particularly, discotic particles with a high aspect ratio are preferred interfacial stabilizers. One of ideal candidates to generate Pickering emulsions is the amphiphilic Janus particle^9^. The Janus name refers the Roman god of portals, Janus, whose two faces look in opposite directions^10^. The two halves of Janus particles possess distinct chemical properties: one half is hydrophobic, the other, hydrophilic. Recently, our group reported a simple protocol to generate Janus and Gemini particles as surfactants to facilitate nanoemulsion generation^8^, where Gemini particle were the platelets with two hydrophilic faces and hydrophobic edges. ZrP, which has a lamellar crystal structure, was grafted with octadecyl isocyanate and then exfoliated with tetrabutylammonium hydroxide (TBA^+^OH^−^), to produce ZrP-C18 platelets. The rigid Janus and Gemini particles provided robust support for the inner paraffin.

Only a few encapsulated PCMs via Pickering emulsification have been reported^11^, but PCMs encapsulated by Janus and Gemini Pickering emulsion was rarely studied. In previous Pickering encapsulation study, high energy input, such as probe sonication, was needed to produce stable emulsions in a similar way that conventional emulsions are made, due to the energy barrier between the particle suspended in the aqueous solution and the water-oil interface^8^,^11^,^12^. Here, a simple two-step Pickering emulsification process by surface-modified ZrP platelets was performed to produce the NEPCMs. The entire procedure is shown in Figure 1. The low interfacial energy introduced by our modified ZrP particles can stabilize nanoemulsions and enable a narrow size distribution^8^. Also, due to the low interfacial energy, we can generate the alkane nanoemulsions easily by manually shaking the suspensions. The low energy input requirement suggests the feasibility of applying this method to mass production of the NEPCMs for commercial application.

## Results

The pristine ZrP particles produced via the reflux synthesizing method are less regularly hexagonal than the ZrP particles prepared by the hydrothermal method^13^. Figure 2 shows the morphologies for numerous and single ZrP particles using scanning electron microscopy (SEM) and transmission electron microscopy (TEM), respectively. In our previous study, we found that the ZrP monolayer after exfoliation has a thickness of about 0.68 nm^14^. With that information, we estimated there are about 30 layers in each pristine ZrP particle.

The ZrP particles are naturally hydrophilic due to the -OH groups on the surface. The surface modification procedure would enhance hydrophobicity of the particles. During modification^8^, a hydrophobic coupling agent, octadecyl isocyanate (ODI), is grafted onto the exposed edges and flat surfaces of the ZrP crystals. After exfoliation with TBA^+^OH^−^ of ZrP layer crystallites, we would obtain two Janus monolayers, each with a hydrophobic surface and a hydrophilic surface, and about 28 Gemini monolayers, each with a hydrophobic edge and two hydrophilic surfaces. Both Janus and Gemini monolayers can act as surfactants to stabilize Pickering emulsions^8^.

With a small amount (0.024% wt to the aqueous phase) of the ZrP Janus and Gemini monolayers, nonadecane in water (Oil/Water) emulsions were generated by 30 seconds of gentle manual shaking. No high energy input (i.e. sonication) was required. A laser scanning confocal microscope was used to characterize the morphology of the nonadecane/water emulsion. The nonadecane, however, easily solidified at room temperature when one small drop of the emulsion solution was loaded onto a glass slide before the observation under microscope. As a result, we were imaging the solidified nonadecane/water emulsion, in the other words, nonadecane droplets, under the confocal microscope (laser scanning confocal microscope Nikon A1R). Figure 3 shows the microscopic image of the nonadecane droplets, the size of which was quite uniform, 524 ± 91 nm, as measured by dynamic light scattering measurement (DLS, Malvern Instruments Ltd. England). When we were performing the two-step Pickering emulsification, however, the nonadecane was still liquid since a small amount of monomer styrene and initiator AIBN were added into a bulk emulsion solution right after the first manual shaking, cooling the solution only slightly, but the bulk temperature of the solution remained above the melting temperature of nonadecane.

**Figure 1:**
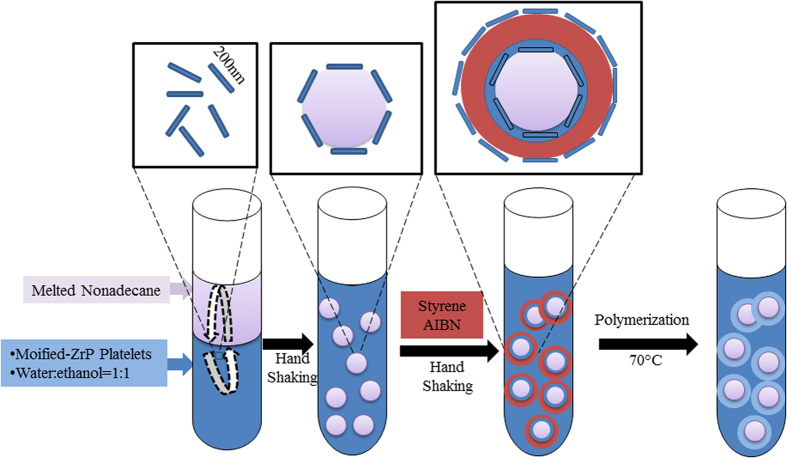
Schematic of NEPCM fabrication via two-step Pickering emulsification. Pickering O/W emulsions were first produced by simple manual shaking for 30 seconds then the coatings on the encapsulation were fabricated by manual shaking for 30 seconds, causing further polymerization into a shell at high temperature.

**Figure 2:**
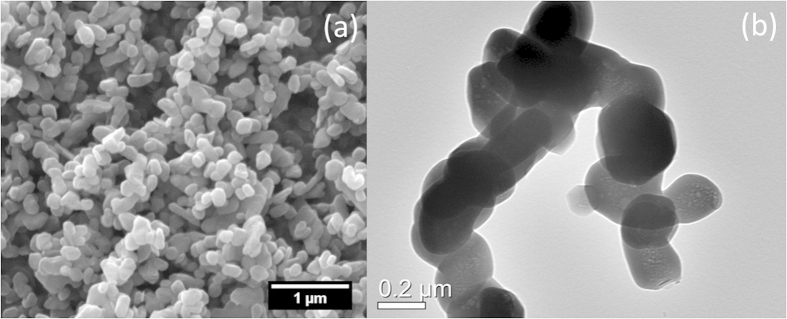
The SEM (**a**) and TEM (**b**) images of ZrP as prepared. The particle sizes range from 200 to 300 nm, and the thickness of the crystallites is roughly 20 nm.

**Figure 3:**
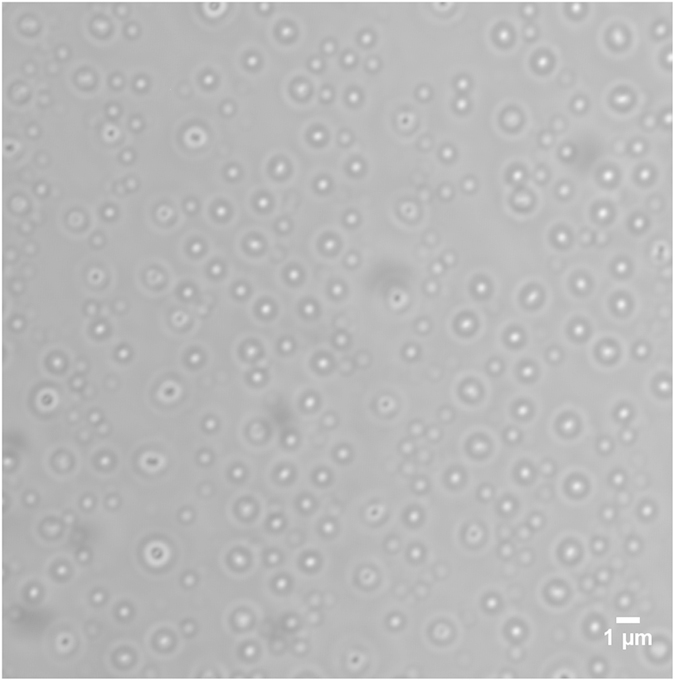
Microscopic image of nonadecane/water droplets by confocal microscope. The emulsion droplets with a light halo were out of focus.

**Figure 4:**
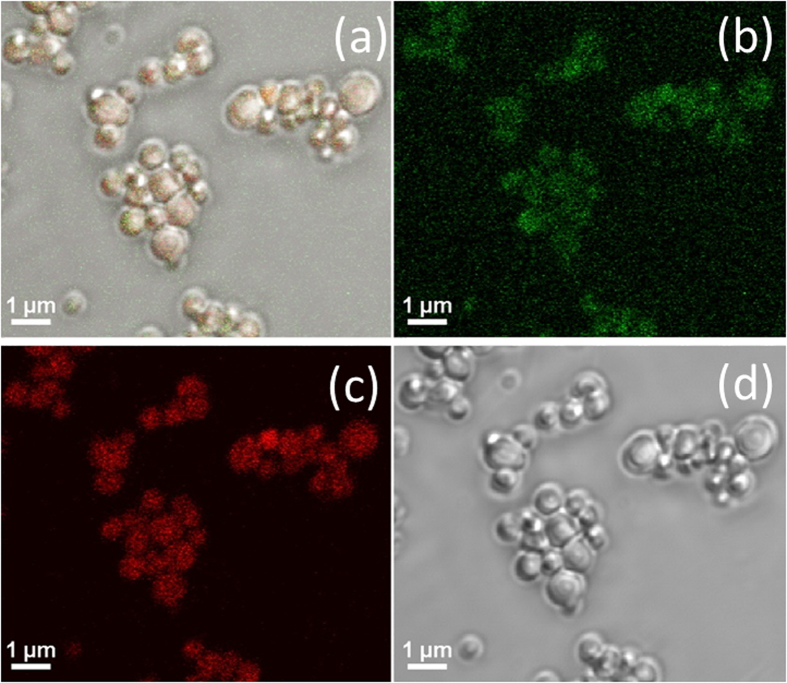
Confocal image of NEPCMS. (**a**) Double fluorescent labelling indicates locations of aqueous phase (fluorescein isothiocyanate, FITC) and nonadecane (Nile Red), (**b**) green channel showing FITC fluorescein fluorescence, (**c**) red channel showing Nile red fluorescence and (**d**) white light showing the NEPCMs without fluorescence.

**Figure 5:**
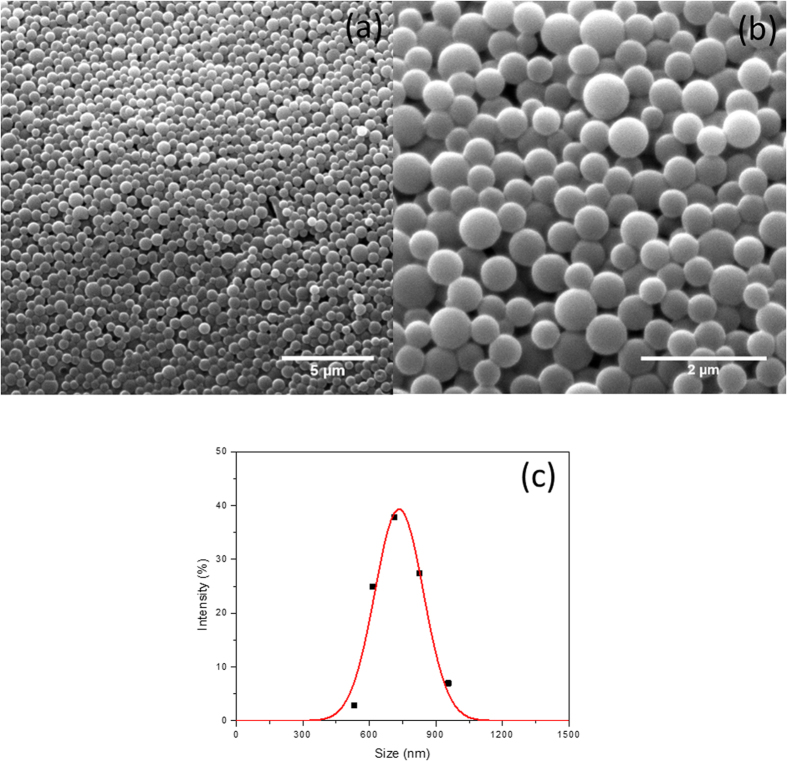
Characterization of NEPCMs. (**a,b**) SEM images of NEPCMs particles, (**c**) size distribution for NEPCMs as measured by DLS.

After the second Pickering emulsification, manual shaking gently again for 30 seconds after addition of monomer and initiator, nonadecane/water/styrene/water multiple emulsions were obtained. The confocal images of the NEPCMs before drying, as shown in Figure 4, indicated the multiple structure of the NEPCMs. The green color came from the fluorescein isothiocyanate (FITC) the aqueous phase while the red color came from the Nile red in the nonadecane. Both colors showing together in the image meant there were a small water layer between the nonadecane and PS after the NEPCMs were fabricated. This assumption could be explained by the emulsion stabilization mechanism as well. Hence, we proposed a schematic for the two-step Pickering emulsification procedure. As shown in Figure 1, the nonadecane-in-water emulsions together with the hydrophobic initiator would be the oil phase for the second step emulsification. By the polymerization of styrene at 70 °C, a core-shell structure was created in which the phase changing material nonadecane was the core and the polystyrene was the shell. Figure 5a,b show the SEM image of NEPCMs (TEM images are available in Supplementary Information Figure S1). They were relatively uniform, as shown in Figure 5c: the size of these NEPCMs was measured by DLS to be 734 ± 110 nm. When we used more materials to synthesize NEPCMs, in a 250 mL reusable glass media bottle, we were able to produce 10g NEPCMs with similar quality (as shown in Supplementary Information Figure S2).

We know that the thickness of the shell layer of NEPCMs was about 110 nm by simple calculation. There are two advantages to the two-step emulsification to produce NEPCMs. First, only a small amount of energy is required for the emulsification procedure. The ability to produce Pickering emulsions using low energy input is due to decreased surface tension in the water phase caused by the addition of ZrP-C18 nanoplates^8^ (Supplementary Information Figure S3) and the co-solvent ethanol together. Moreover, the size of either nonadecane/water emulsion or NEPCMs was quite uniform. Employing modified ZrP, rather than molecular surfactants, promotes formation of more stable Pickering emulsion due to the limited coalescence in the solid-stabilized emulsions as described by Arditty^15^. The nanoplates absorbed on the water-oil interface prevents coalescence of Pickering emulsion droplets and, therefore, hinders creation of larger emulsion droplets. Prevention of coalescence, in other words, promotes formation NEPCMs with uniform size distribution.

**Figure 6:**
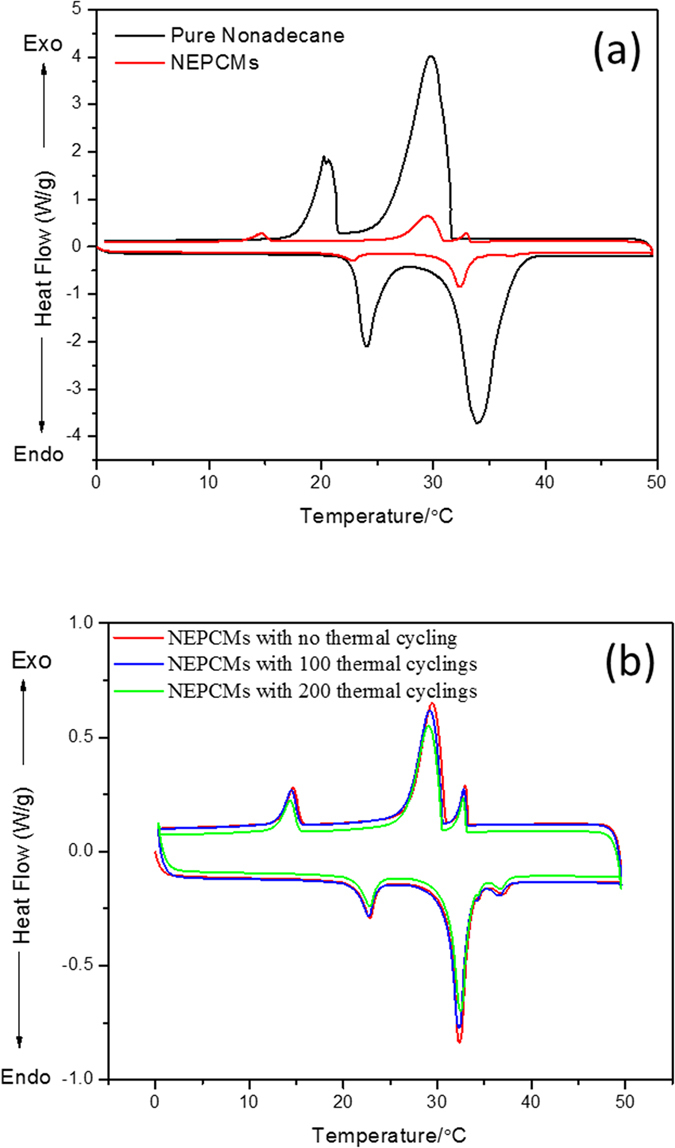
Thermal property and stability of NEPCMs. (**a**) DSC traces of pure nonadecane and NEPCMs during the heating-cooling processes, the NEPCMs specimen was held first at 0 °C for 2 minutes, then heated from 0 °C to 50 °C at a rate of 5 °C/min, and finally cooled to 0 °C at a controlled rate of 5 °C/min. (**b**) DSC traces of NEPCMs after 100 and 200 heating-cooling cycles. The first 100 cycles were performed at a fast heating-cooling rate of 3 °C/s. The heating-cooling rates for the second 100 cycles followed those used for DSC measurement, 5 °C/min heating to 50 °C and then 5 °C/min cooling to 0 °C, staying at both 0 °C and 50 °C for 30 seconds.

The thermal behavior of the NEPCMs was measured using differential scanning calorimetry (DSC, TA Instruments, United States) under normal nitrogen conditions. Heat flow versus temperature was obtained via DSC characterization, with results shown in Figure 6a. As temperature increased from 0 to 50 °C, there were two endothermic peaks for pure nonadecane at 24.00 °C and 34.12 °C. The first endothermic peak at 24.00 °C was due to the rotator phase of n-alkanes below the melting temperature^13^. The second endothermic peak at 34.12 °C indicates the melting temperature. In the cooling process, two exothermic peaks were obtained. The crystallization point was 29.79 °C, and the nonadecane was converted to stable crystal phase at 20.25 °C, at which temperature it was trapped in the face-centered orthorhombic rotator phase^16^.

As for the NEPCMs, however, their thermal behavior was quite different (Figure 6a,b). Three endothermic peaks appeared in the heating process, 22.77 °C, 32.30 °C, and 36.95 °C. The peak at 22.77 °C was attributed to the change from orthorhombic crystal to rotator phase, and the peaks at 32.30 °C and 36.95 °C were due to the conversion from rotator phase to metastable phase and metastable phase to liquid phase, respectively. Three exothermic peaks appeared in the cooling process, 32.90 °C, 29.47 °C and 14.67 °C. The peak at 32.90 °C was attributed to the exothermic peak for nonadecane transitioning from isotropic liquid to new metastable phase. The big exothermic peak at 29.47 °C was caused by the transition from metastable phase to rotator phase; at 14.67 °C, the rotator phase became orthorhombic crystal.

Comparing the DSC thermal behaviors of the NEPCMs to that of pure nonadecane, we found that there was one additional peak in both heating and cooling procedure for NEPCMs than occurred for pure nonadecane. For example, there was a new endothermic peak at 36.95 °C in the heating procedure and a new exothermic peak at 32.90 °C in cooling procedure. The new peaks were caused by the surface freezing of the encapsulated nonadecane, which caused the encapsulated nonadecane become a metastable phase. Moreover, the positions of either endothermic peaks or exothermic peaks of NEPCMs were delayed slightly compared with those of pure nonadecane. This is normal for most encapsulated phase changing materials, and can be improved by adding better thermal conductivity materials into the shell of encapsulated materials.

Two more series of DSC measurements were carried out on the same NEPCMs sample to obtain the stability of this material (Figure 6b). The thermal cycling was performed in a P × 2 thermal cycle machine (Thermo Electron Corporation, USA). The DSC curve for NEPCMs, after 100 cycles at a fast heating-cooling temperature rate of 3 °C/min, shown as blue line in Figure 6b, almost duplicated the first curve (red line), which is the thermal curve for NEPCMs with no thermal cycle. The green line in Figure 6b shows the DSC curve for the same sample after 100 cycles at exactly the same temperature conditions as for DSC characterization. It was found that the peaks were located at almost the same temperature for the three curves, proving the stability of the NEPCMs. The height of the peak for the 100-cycle curve, however, was slightly lower that the curve depicting no cycling; and similarly, the height of the peak after 200 cycles was slightly lower than that for 100 cycles.

## Discussions

In this paper, the encapsulation efficiency of nonadecane in NEPCMs was determined from the following equation (1)^17^. Encapsulation efficiency determined the amount of PCMs which had been encapsulated over the total PCMs added as material, it was depended on the encapsulation quantity. Less than 100% encapsulation efficiency meant some of the PCMs hadn’t been encapsulated.


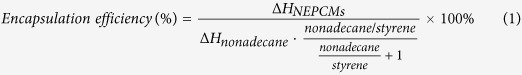


where ΔH was the latent heat of the materials; nonadecane/(styrene) was the mass ratio of core (nonadecane) over shell (styrene) in the equation; and ΔH_nonadecane_ and ΔH_NEPCMs_ were the latent heat of nonadecane and NEPCMs measured by DSC, respectively. So the encapsulation efficiency of NEPCMs mentioned above was 55.9%. The efficiency here was not as high as that of many microencapsulated phase changing materials, but was competitive with some NEPCMs stabilized by molecular surfactants^18^.

The possible reasons for the relatively low encapsulation efficiency were proposed as following: 1) although the NEPCMs size was relatively uniform, there was a possibility that some NEPCMs had higher nonadecane to PS (nonadecane/PS) ratio and tended to cream to the top of the suspension, and these kind of samples would be discarded in the washing procedure. 2) It was also possible that some of the nonadecane/water emulsions were demulsified in the second emulsification and failed to be encapsulated any more, which would lower the encapsulation efficiency as well. In our experiment, when the polymerization was finished and the whole samples were cooled down to room temperature, we did noticed that there were a small layer consisted of NEPCMs and waxy nonadecane on the top of the suspensions, we assumed these NEPCMs had a higher nonadecane/PS ratio.

Here are the suggestions and our future work to improve the encapsulation efficiency: 1) instead of nonadecane, we will switch to another higher density phase change material so that we will be able to avoid the losing of high nonadecane/PS ratio samples. 2) Add some higher density particles into oil phases before emulsification so that the higher nonadecane/PS ratio samples would not cream to the top. 3) Use magnetic stirring, instead of gently manual shaking, for two step emulsification shaking to make emulsion even more uniform. Moreover, we will stir the suspensions during the polymerization instead of just keep them static, which may contribute to the uniformity of the capsules as well. 4) For the scale-up samples, we are thinking about the recycling of the top layer mentioned above, mainly of which are high nonadecane/PS ratio NEPCMs and nonadecane. The top layer will be collected and heated up to 50 °C until nonadecane melt, and then a flirtation will be performed at 50 °C to separate the NEPCMs and liquid nonadecane. And we can do a characterization on these NEPCMs after that. On the other hand, we can reuse the nonadecane so that we don’t waste the material, this is especially important for the industrial application.

In conclusion, we demonstrated for the first time that NEPCMs can be mass-produced via a simple two-step Pickering emulsification procedure. This low-energy procedure requires only gently manual shaking to produce products. The NEPCMs were proved to be thermally stable and uniform in size. A primary result showed that we can make up to 10 g NEPCMs for one batch in the lab, which was limited merely by the capacity of the reaction container. The Pickering emulsification method based on the asymmetric ZrP-C18 nanoplates for fabrication of nanoencapsules allows for a wide range of engineering applications, not only in PCM, but also in cosmetics, drug delivery, and food production.

## Methods

### Synthesis of ZrP

Zirconium phosphate (ZrP) was prepared by the reflux method: 6 g of zirconyl chloride octahydrate (ZrOCl_2_·8H_2_O) was mixed with 50 mL of 12 M phosphate acid. The mixture was loaded in a 100-mL round flask and refluxed for 24 hours using oil bath at 94 °C. The product was washed with deionized water three times, dried in the oven, and stocked for later use. Scanning electron microscopic (FEI Quanta 600 FE-SEM) and transmission electron microscopic (JEOL JEM-2010 TEM) characterizations were used to observe the morphology of the ZrP.

### Preparation of ZrP-C18 Pickering emulsifier

The dried white product was ground into a fine powder with mortar and pestle. Depending upon ambient humidity and the length of time after the ZrP is produced, one more drying process might be preferred to remove the resident water before the surface modification reaction. The crystals were reacted with octadecyl isocyanate in a 1:10 (ODI: ZrP) molar ratio at 90 °C for 24 hours under nitrogen, using extra dry toluene as a solvent. The resulting surface-modified ZrP (as ZrP-C18 later in this paper) was washed with methanol three times and dried overnight in an oven at 70 °C.

The Janus and Gemini ZrP-C18 nanoplates were obtained by exfoliating the crystals into monolayers using tetrabutylammonium hydroxide (TBA^+^OH^−^) at a molar ratio of 1:1 in DI water at room temperature.

### Two-step Pickering emulsification to fabricate NEPCMS

First, 0.02 g nonadecane was melted in 1 mL deionized water, and then mixed with 1 mL ethanol co-solvent and 100 μL ZrP-C18 suspension (containing 5% wt of ZrP-C18). The mixture was gently shaken for 30 seconds, which produced the nonadecane in water (oil in water, O/W) emulsions for the next step. In the second step, 100 μL styrene monomer together with 0.0016 g initiator azobisisobutyronitrile (AIBN) were added into the O/W emulsions produced in the first step, and the new mixture was manually shaken for another 30 seconds. Finally, the entire NEPCMs precursor was transferred into a 70 °C oven for 4 hours, allowing for the full polymerization of styrene, with polystyrene as the shell. The NEPCMs sample were raised with warm DI water (50 °C) for twice and then dry using a freezing dry machine.

For the scale-up test, we used 100 times more materials: 4 g nonadecane, 20 mL styrene, 20 mL ZrP-C18 suspension and 100 mL DI water and 100 mL ethanol, and followed the two-step Pickering emulsification procedure.

## Additional Information

**How to cite this article**: Wang, X. *et al.* Nano-encapsulated PCM via Pickering Emulsification. *Sci. Rep.* **5**, 13357; doi: 10.1038/srep13357 (2015).

## Supplementary Material

Supplementary Information

## References

[b1] Sabbah R., Farid M. M. & Al-Hallaj S. Micro-channel heat sink with slurry of water with micro-encapsulated phase change material: 3D-numerical study. Appl. Therm.Eng. 29, 445–454 (2009).

[b2] Jamekhorshid A., Sadrameli S. M. & Farid M. A review of microencapsulation methods of phase change materials (PCMs) as a thermal energy storage (TES) medium. Renew. Sust. Energ. Rev. 31, 531–542 (2014).

[b3] Peippo K., Kauranen P. & Lund P. D. A Multicomponent PCM wall optimized for passive solar heating. Enery Build. 17, 259–270 (1991).

[b4] Hawlader M. N. A., Uddin M. S. & Khin M. M. Microencapsulated PCM thermal-energy storage system. Appl. Energ. 74, 195–202 (2003).

[b5] Kuznik F., David D., Johannes K. & Roux J.-J. A review on phase change materials integrated in building walls. Renew. Sust. Energ. Rev. 15, 379–391 (2011).

[b6] Shin Y., Yoo D. I. & Son K. Development of thermoregulating textile materials with microencapsulated phase change materials (PCM). IV. Performance properties and hand of fabrics treated with PCM microcapsules. J. Appl. Poly. Sci. 97, 910–915 (2005).

[b7] Vignati E., Piazza R. & Lockhart T. P. Pickering emulsions: Interfacial tension, colloidal layer morphology, and trapped-particle motion. Langmuir 19, 6650–6656 (2003).

[b8] Mejia A. F. *et al.* Pickering emulsions stabilized by amphiphilic nano-sheets. Soft Matter 8, 10245–10245 (2012).

[b9] Walther A. & Muller A. H. E. Janus particles. Soft Matter 4, 663–668 (2008). 10.1039/b718131k 32907169

[b10] Degennes P. G. Soft Matter (Nobel Lecture). Angew. Chem. Int. Ed. 31, 842–845 (1992).

[b11] Schrade A., Cao Z., Landfester K. & Ziener U. Preparation of raspberry-like nanocapsules by the combination of pickering emulsification and solvent displacement technique. Langmuir 27, 6689–6700 (2011). 21563812 10.1021/la201170w

[b12] Zhang K., Wu W., Meng H., Guo K. & Chen J. F. Pickering emulsion polymerization: Preparation of polystyrene/nano-SiO_2_ composite microspheres with core-shell structure. Powder Technology 190, 393–400 (2009).

[b13] Shuai M., Mejia A. F., Chang Y.-W. & Cheng Z. Hydrothermal synthesis of layered α-zirconium phosphate disks: control of aspect ratio and polydispersity for nano-architecture. CrystEngComm 15, 1970–1977,(2013).

[b14] He P. *et al.* Hindrance function for sedimentation and creaming of colloidal disks. Phys.Rev. E 81, 026310 (2010). 10.1103/PhysRevE.81.026310 20365654

[b15] Arditty S., Whitby C. P., Binks B. P., Schmitt V. & Leal-Calderon F. Some general features of limited coalescence in solid-stabilized emulsions. Eur. Phys. J. E 12, 355–355 (2003). 10.1140/epje/i2003-10018-6 15011047

[b16] Xie B. *et al.* Crystallization behaviors of n-nonadecane in confined space: Observation of metastable phase induced by surface freezing. J. Phys. Chem. B 110, 14279–14282 (2006). 16854133 10.1021/jp063201j

[b17] Sánchez-Silva L., Tsavalas J., Sundberg D., Sánchez P. & Rodriguez J. F. Synthesis and characterization of paraffin wax microcapsules with acrylic-based polymer shells. Ind. Eng. Chem. Res. 49, 12204–12211 (2010).

[b18] Fang Y., Yu H., Wan W., Gao X. & Zhang Z. Preparation and thermal performance of polystyrene/n-tetradecane composite nanoencapsulated cold energy storage phase change materials. Energy Convers. Manage. 76, 430–436 (2013).

