# Supplementary material for: Nano-encapsulated PCM via Pickering Emulsification
Source: Sci Rep. 2015 Aug 17;5:13357. doi: 10.1038/srep13357 (PMC4538601; doi:10.1038/srep13357)
Supplement: Supplementary Information [file srep13357-s1.doc]

**Nano-encapsulated PCM via Pickering Emulsification**

Xuezhen Wang^ab^, Lecheng Zhang^ab^, Yi-Hsien Yu^c^, Lisi Jia^d^, M. Sam Mannan^abc^, Ying Chen^d^, Zhengdong Cheng*^abcde^

^a^ Artie McFerrin Department of Chemical Engineering, Texas A&M University, College Station, TX, 7843-3122, USA

^b^ Mary Kay O'Connor Process Safety Center, Artie McFerrin Department of Chemical Engineering, Texas A&M University, College Station, TX, 77843-3122, USA

^c^ Department of Materials Science and Engineering, Texas A&M University, College Station, TX, 77843-3003, USA

^d^ Soft Matter Center, Guangdong Province Key Laboratory on Functional Soft Condensed Matter, School of Materials and Energy, Guangdong University of Technology, Guangzhou, 510006, China

^e^ Professional Program in Biotechnology, Texas A&M University, College Station, TX, 77843-3122, USA

1. TEM image of NEPCMs

(a)


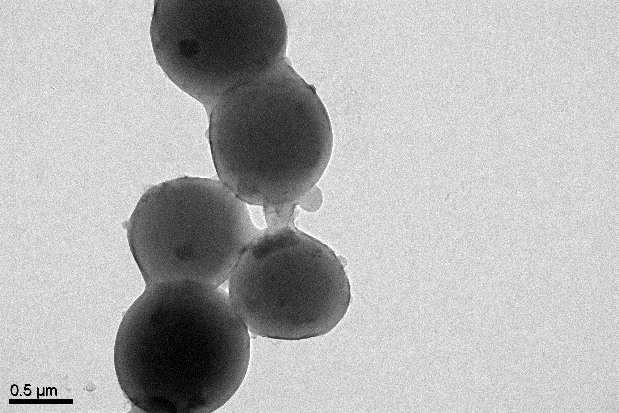

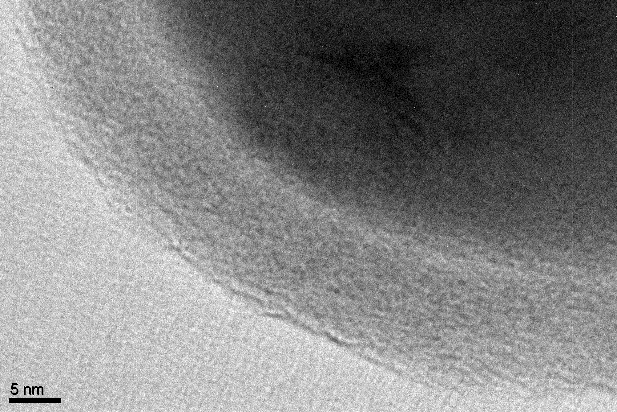


(b)

**Figure S1 TEM images for NEPCMs.**

1. Mass production of NEPCMs

(a)


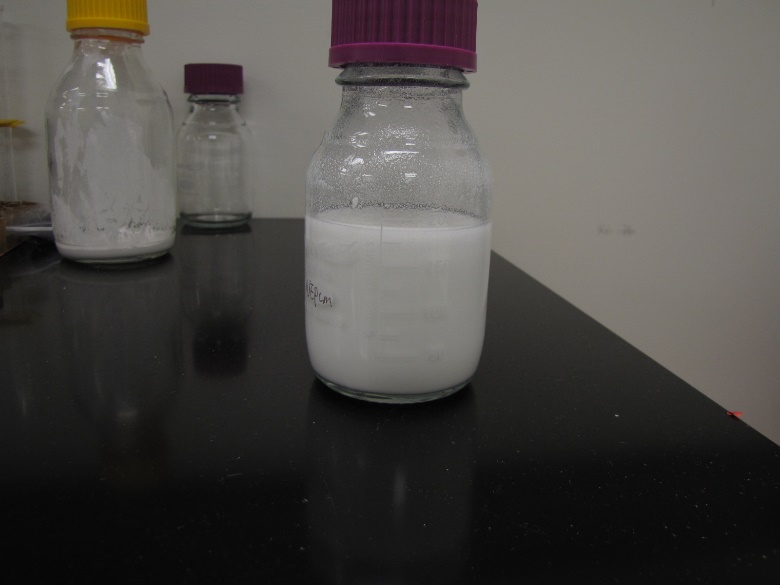

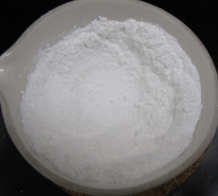


(b)

**Figure S2 Mass production of 10g NEPCMs.** A) Right after prepared, b) after drying.

1. Surface tension of ZrP-C18

The surface tension of the ZrP-C18 was measured by Wilhelmy plate (L-B-small, NIMA UK) method.

**Figure S3 Surface tension as a function of ZrP-C18 concentration.**
